# Methane-Rich Saline: A Potential Resuscitation Fluid for Hemorrhagic Shock

**DOI:** 10.1155/2019/4929107

**Published:** 2019-11-26

**Authors:** Yingmu Tong, Yanyan Dong, Yang Feng, Zeyu Li, Yifan Jia, Xing Zhang, Kai Qu, Chang Liu, Jingyao Zhang

**Affiliations:** ^1^Department of Hepatobiliary Surgery, The First Affiliated Hospital of Xi'an Jiaotong University, Xi'an Shaanxi 710061, China; ^2^Department of Immunology and Laboratory, Shaanxi University of Chinese Medicine, Xianyang Shaanxi 712046, China; ^3^Department of SICU, The First Affiliated Hospital of Xi'an Jiaotong University, Xi'an Shaanxi 710061, China

## Abstract

Hemorrhagic shock is caused by massive blood loss. If the patient is not fully resuscitated in time, this may eventually lead to multiple organ failure and even death. Previous studies on methane-rich saline in animal models showed that it confers resistance against many diseases. In this study, we explored the protective effect of methane-rich saline, used as a resuscitation fluid, in hemorrhagic shock. Hemorrhagic shock was induced in SD rats by bloodletting via intubation of the right femoral artery. The rats were divided into three groups: a sham control group (sham control), a shock group resuscitated by an infusion of autologous blood and an equivalent volume of normal saline (Shock+NS), and a shock group resuscitated by an infusion of autologous blood and an equivalent volume of methane-rich saline (Shock+MRS). Assessment of blood pressure and levels of plasma lactate showed that resuscitation using methane-rich saline (MRS) restored systemic blood pressure and reduced the levels of lactate in the plasma. Meanwhile, lower levels of serum IL-6 and TNF-*α* were also observed in the group resuscitated with MRS. In the heart, liver, and kidney, MRS reduced inflammation and oxidative stress levels. Analysis of organ function via levels of biochemical indicators revealed that the group resuscitated with MRS had reduced serum levels of AST and CK, indicating a potential cardioprotective effect. The expression levels of apoptosis-related proteins, including those of Bcl-2/Bax, and the results of TUNEL-labeling assay indicated that MRS significantly reduced apoptosis in the heart. Methane also had a positive effect on the expression of the PGC-1*α*/SIRT3/SOD2 signaling pathway. Our results showed that MRS can potentially serve as a novel resuscitation fluid because of its anti-inflammatory, antioxidative, and antiapoptotic properties.

## 1. Introduction

Hemorrhagic shock is a physiological condition accompanied by rapid blood loss. It is characterized by severe vasoconstriction, insufficient tissue perfusion, and cellular hypoxia. In response to decreased oxygen tension, oxidative phosphorylation decreases and anaerobic metabolism increases in an attempt to maintain the cellular energy [1]. Without rapid and adequate resuscitation, hemorrhagic shock can develop, progressing from cellular dysfunction to organ failure and eventually leading to death [2]. Approximately 1.9 million people worldwide die from hemorrhagic shock every year, and those who survive the initial hemorrhagic shock show poor functional recovery and significantly increased long-term mortality [3].

Methane, one of the most abundant organic greenhouse gases found in the atmosphere, is detected in 30-50% of healthy adults worldwide [4]. Methane has garnered increasing attention as a potential therapeutic against various diseases. Studies indicate that methane possesses important biological activity that can protect cells and organs from inflammation, oxidative stress, and cellular apoptosis [5]. Previous studies have shown that methane-rich saline (MRS) plays a protective role in animal models of myocardial ischemia [6] and in ischemia-reperfusion injury of the liver [7] and kidney [8] by exerting antioxidant, anti-inflammatory, and antiapoptotic effects. Therefore, we hypothesized that MRS can be used as a new resuscitation fluid to treat patients with hemorrhagic shock and protect against organ damage. In this study, we explored the therapeutic effects of MRS in a rat model of hemorrhagic shock and examined the potential molecular mechanisms involved.

## 2. Materials and Methods

### 2.1. Animals and Experimental Procedures

Healthy male SD rats (250–300 g, Animal Feeding Center of Xi'an Jiaotong University Health Science Center) were housed with constant temperature, humidity, and a timed 12-hour light/dark cycle. Animals were allowed to acclimate for at least 3 days before experiments. The animal experiment was performed in accordance with the Guide of Laboratory Animal Care and Use from the United States National Institutes of Health and was approved and supervised by the Institutional Animal Care and Use Committee of the Ethics Committee of Xi'an Jiaotong University Health Science Center, China (IACUC protocol number: XJTULAC2014-207).

The rats were anesthetized by intraperitoneal injection of sodium pentobarbital (50 mg/kg). The bilateral femoral artery and right femoral vein were catheterized by PE50 tubing for blood pressure measurement (MAP), blood collection, and input of resuscitation fluid. MAP was recorded using the experimental protocol BL-420F from Data Acquisition & Analysis System. Hemorrhagic shock was induced by bloodletting via catheter of the right femoral artery. MAP reached 30 mmHg in approximately 15 minutes. The outflow blood was collected into a sterile syringe containing 10 units of heparin and kept at 37°C before reinfusion. If MAP exceeded 35 mmHg, it was reduced to, and maintained at, 30 + 5 mmHg by additional bloodletting. If MAP fell below30 mmHg, autologous blood was infused to maintain the MAP. MAP was then maintained at 30 + 5 mmHg for 1 hour. Then, within the subsequent 15 minutes, the rats were resuscitated by an infusion of all collected autologous blood and resuscitation solution (1 : 1) to restore blood pressure; normal blood pressure was maintained for 2 hours. The Shock+NS group (*n* = 9) is a shock group resuscitated by an infusion of autologous blood and an equivalent volume of normal saline. The Shock+MRS group (*n* = 9) is a shock group resuscitated by an infusion of autologous blood and an equivalent volume of methane-rich saline. The sham control group (*n* = 9) received only anesthesia and intubation but no intervention.

After 2 hours of resuscitation, the rats were anesthetized with sodium pentobarbital (50 mg/kg). Blood and heart, liver, and kidney tissues were rapidly collected, and rats were euthanized under anesthesia.

### 2.2. Methane-Rich Saline Preparation

Methane was dissolved in sealed normal saline and underwent high pressure (0.4 MPa) for 8 hours to produce MRS. Prepared MRS was stored in an aluminum bag under atmospheric pressure at 4°C and sterilized by *γ*-radiation one day before utilization. The concentration of MRS was detected using gas chromatography (Shanghai Qiyang Standard Gas Ltd., Shanghai, China) as previously described [9]. According to calculation, the concentration of the MRS was 1.2~1.5 mmol/L.

### 2.3. Histologic Analysis

Two hours after resuscitation and 3.5 hours after sham operations, the cardiac, liver, and kidney tissue samples were assessed with hematoxylin and eosin (H&E) staining. The tissues were fixed in 10% formalin for 48 hours and then treated with paraffin embedding. Serial sections of 4 *μ*m thickness were obtained and stained with hematoxylin and eosin (H&E). Then, two researchers assessed the histologic changes in a double-blinded way to select a representative field for future use through the light microscope. And the investigators were looking for the edema, necrosis, and inflammatory cell immersion tissue areas. The degree of edema, necrosis, and inflammatory cell immersion was expressed as the mean of twelve different fields within each slide classified on a scale of 0-3 (normal-0, mild-1, moderate-2, and severe-3). And the total histopathological score is the sum of the degree of edema, necrosis, and inflammatory cell immersion.

### 2.4. Blood Chemistries and Inflammatory Cytokines

Lactic acid, blood urea nitrogen (BUN), serum creatinine (Cr), aspartate aminotransferase (AST), alanine aminotransferase (ALT), and creatinine kinase (CK) were determined by commercial kits (Jiancheng Institute of Biotechnology, Nanjing, China). IL-6 and TNF-*α* were determined by ELISA (NeoBioscience, Shenzhen, China).

### 2.5. Quantitative Real-Time PCR

We followed the methods of Sims et al. [10]. RNA from the heart, liver, and kidney was extracted from frozen tissue using TRIzol (MilliporeSigma) with ethanol precipitation. According to the manufacturer's recommendation, RNA (1 *μ*g) was used to produce the cDNA using the High-Capacity cDNA Reverse Transcription Kit (Applied Biosystems). Real-time PCR was performed using Applied Biosystems 7900HT (Applied Biosystems) with SYBR green. Two technical duplications were obtained for each sample, actin was used as housekeeping gene, and the relative expression level of RNA was calculated by using the *ΔΔ*CT method. The following primers were used: *β*-actin (forward, GGCTGTATTCCCCTCCATCG; reverse, CCAGTTGGTAACAATGCCATGT), IL-6 (forward, TAGTCCTTCCTACCCCAACTTCC; reverse, TTGGTCCTTAGCCACTCCTTC), and TNF-*α* (forward, CTGTGCCTCAGCCTCTTCTC; reverse, ACTGATGAGAGGGAGCCCAT).

### 2.6. Oxidation Index Detection

The levels of MDA in heart, liver, and kidney tissue were measured by commercial biochemical kits (Jiancheng Institute of Biotechnology, Nanjing, China) following the manufacturer's instructions, and the activities of SOD in cardiac, liver, and kidney tissue were measured by commercial biochemical kits (Beyotime Biotechnology, Shanghai, China).

### 2.7. Western Blot Assay

For western blot analysis, frozen cardiac, liver, and kidney tissues were lysed in RIPA buffer supplemented with phosphatase inhibitors and protease inhibitors using a tissue lyser. Lysates were centrifuged at 14000g for 15 minutes at 4°C. Lysates were denatured in 25% Laemmli buffer+BME at 95°C for 10 minutes and were separated using sodium dodecyl sulfate-polyacrylamide gel electrophoresis (SDS-PAGE). Then the proteins were transferred onto polyvinylidene difluoride (PVDF) membranes. The resulting blots were blocked with 8% skim milk and incubated with anti-Bax antibody (1 : 1000, Sanying Biotechnology, China), anti-Bcl-2 antibody (1 : 1000, Sanying Biotechnology, China), anti-PGC-1*α* antibody (1 : 2000, Sanying Biotechnology, China), anti-SIRT3 antibody (1 : 1000, Sanying Biotechnology, China), anti-SOD2 antibody (1 : 1000, Sanying Biotechnology, China), anti-Ac-SOD2 antibody (1 : 1000, Abcam, USK), and anti-*β*-actin antibody (1 : 10000, Santa Cruz Biotechnology, USA) overnight at 4°C. Subsequently, the bolts were washed three times with PBS and incubated with anti-rabbit and anti-mouse horseradish peroxidase-conjugated secondary antibodies (1 : 10000, Abmart, China) for 1 h at 37°C. Proteins of interest were detected by Western Lightning Plus-ECL (Perkin Elmer). Images were captured using a Bio-Rad imaging station and quantified using ImageJ.

### 2.8. Apoptosis Assay

Two hours after resuscitation, cardiac tissue was assessed with TUNEL staining with a fluorescence detection kit (In Situ Cell Death Detection Kit; Fluorescein, Roche, Switzerland). The sections were observed with a fluorescence microscope, and representative fields were chosen for application.

### 2.9. Statistical Analysis

Results are expressed as mean ± SEM. Comparison between 2 groups was performed using a 2-tailed Student's *t* or Mann-Whitney test, depending on normality of data distribution. Two-way ANOVA was used to look at changes over time between groups. One-way ANOVA was used to compare 3 or more groups with a post hoc 2-tailed Student's *t* or Mann-Whitney test if statistically significant (*P* < 0.05). All statistical analysis was performed using Prism7 (GraphPad Software Inc.), with *P* < 0.05 considered statistically significant.

## 3. Results

### 3.1. Resuscitation with Methane-Rich Saline Reduced Lactic Acidosis in the Fixed-Pressure Hemorrhagic Shock Rat Model

**Figure 1:**
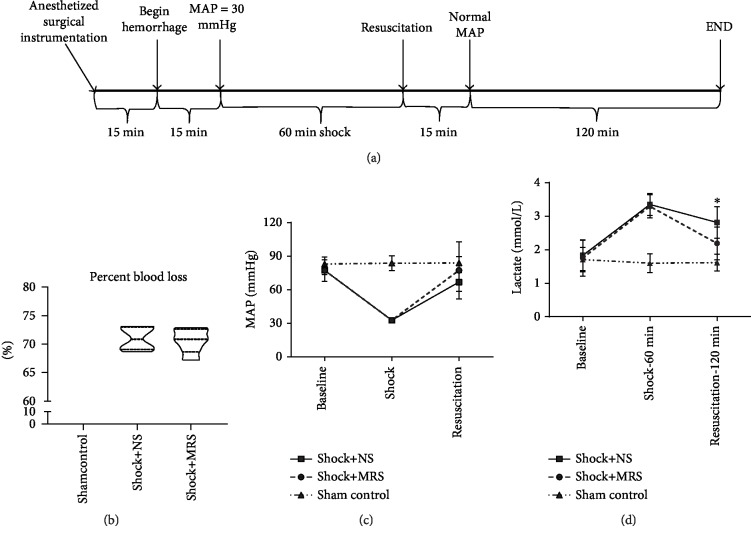
Experimental design and physiologic variables. (a) Animals were bled to obtain a mean arterial blood pressure (MAP) of 30 mmHg for 60 minutes and then resuscitated with two times the shed volume using methane-rich saline/normal saline and blood (1 : 1) for 15 minutes and for the remaining 120 minutes. (b) NS-treated animals did not differ from MRS-treated animals in terms of percentage of total blood volume shed. (c) MAP was similar between the groups. (d) MRS-treated animals showed significantly lower lactate levels at 120 minutes after resuscitation. ^∗^*P* < 0.05 Shock+NS group versus Shock+MRS group.

To investigate the physiologic effect of using methane-rich saline (MRS) in resuscitation from hemorrhagic shock, we generated a fixed-pressure hemorrhagic shock rat model. We then utilized this model to assess resuscitation with autologous blood and MRS or normal saline (NS) (Figure 1(a)). The volume of resuscitation fluid was two times the volume of outflow blood during hemorrhagic shock. The Shock+MRS group and the Shock+NS group were maintained at a mean arterial blood pressure (MAP) of 30 + 5 mmHg for 60 minutes. Both groups had a similar baseline MAP and percentage of total blood volume shed (Figure 1(b)), and blood pressure post resuscitation was statistically not indistinguishable between the groups (Figure 1(c)). Importantly, the rats resuscitated with MRS had a significantly lower level of serum lactate at 120 minutes after resuscitation (Figure 1(d)).

### 3.2. The Effect of MRS on Organ Injury and Organ Function

**Figure 2:**
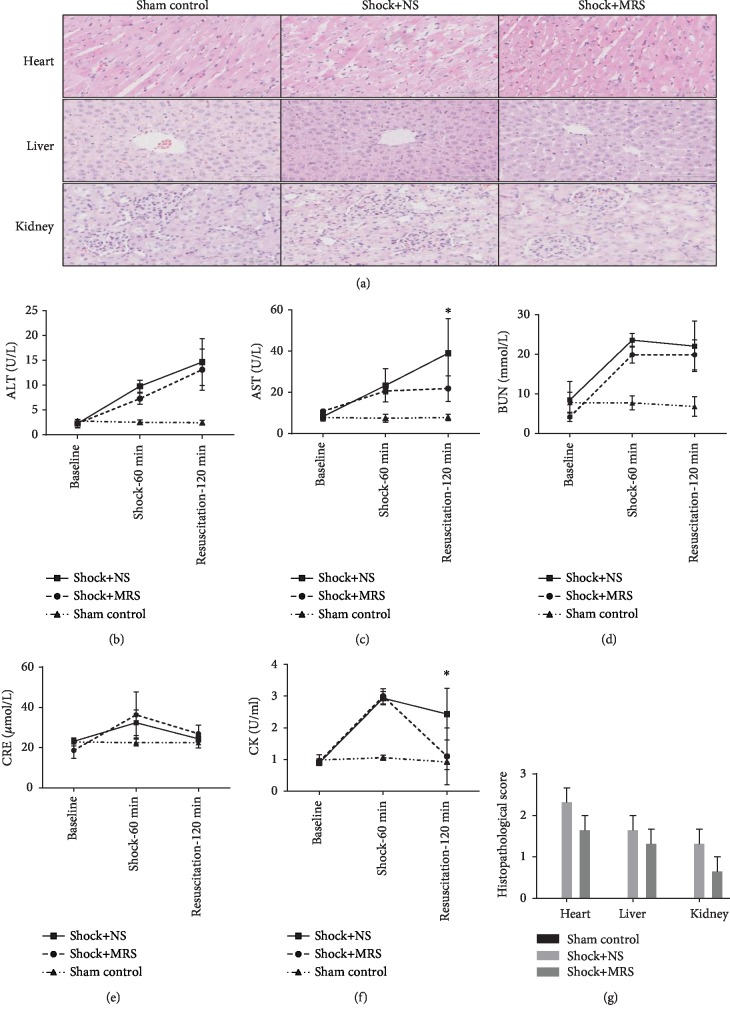
Representative histology photomicrographs and organ function. (a) H&E staining was performed on liver, kidney, and cardiac tissue sections. Representative sections; original magnification, 200x. (b) Serum alanine aminotransferase activity. (c) Serum aspartate aminotransferase activity. (d) Serum blood urea nitrogen level. (e) Serum creatinine level. (f) Serum creatine kinase activity. (g) Histopathological score. ^∗^*P* < 0.05 Shock+NS group versus Shock+MRS group.

At 120 minutes after resuscitation, histological analysis revealed that both shock groups showed liver, kidney, and cardiac injury. However, there were no significant organ injuries in both groups except the Shock+NS group, which just had a more obvious edema (Figures 2(a) and 2(g)). To determine whether the administration of MRS preserved organ function, blood samples were collected from the shocked rats at the beginning, 60 minutes after induction of shock, and 120 minutes after resuscitation; these blood samples were assayed for the expression of markers signifying liver and kidney dysfunction. Although induction of hemorrhagic shock and resuscitation generated a significantly increased expression of markers of liver and kidney damage, the values remained within the normal range because of the early time point examined (Figures 2(b)–2(e)). However, at 120 minutes post resuscitation, the Shock+MRS group showed reduced activity of aspartate aminotransferase (Figure 2(c)) but no difference in alanine aminotransferase (Figure 2(b)), which is likely associated with cardiac injury. The activity of creatine kinase, another marker of cardiac injury, was rescued by treatment with MRS (Figure 2(f)). These results indicate that MRS exerted cardioprotective effects that warrant further investigation using this model.

### 3.3. Anti-Inflammation Effects of Methane-Rich Saline on Hemorrhagic Shock and Resuscitation

**Figure 3:**
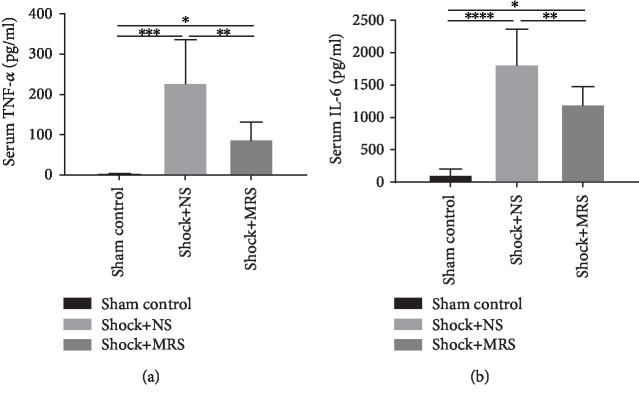
Methane-rich saline suppressed inflammation response caused by hemorrhagic shock and resuscitation. The serum (a) TNF-*α* and (b) IL-6 levels were assessed by ELISA. ^∗^*P* < 0.05, ^∗∗^*P* < 0.01, ^∗∗∗^*P* < 0.001, and ^∗∗∗∗^*P* < 0.0001.

**Figure 4:**
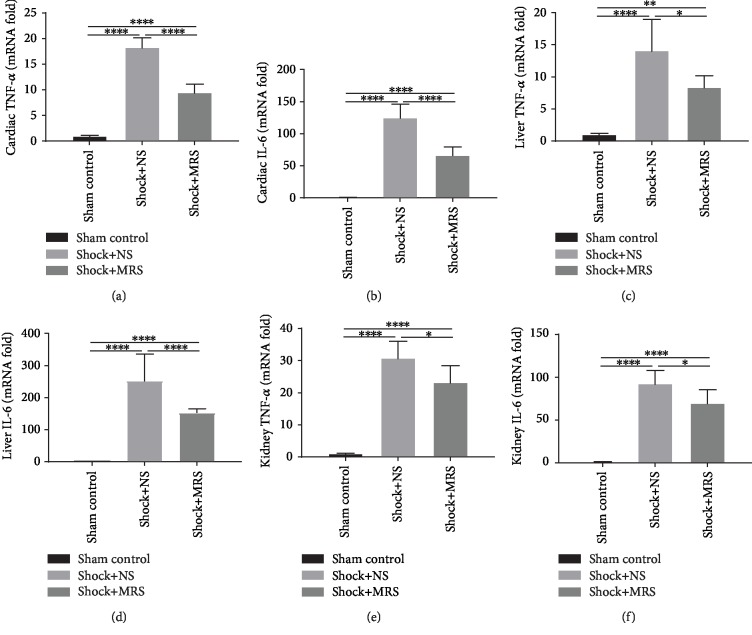
Methane-rich saline suppressed the transcription level of inflammatory cytokines. mRNA levels of Cardiac (a) TNF-*α*, (b) IL-6, Hepatic (c) TNF-*α*, (d) IL-6,renal (e) TNF-*α* and (f) IL-6 was evaluated by qPCR. ^∗^*P* < 0.05, ^∗∗^*P* < 0.01, ^∗∗∗^*P* < 0.001, and ^∗∗∗∗^*P* < 0.0001.

We detected the levels of proinflammatory cytokines in serum samples from the different groups, including IL-6 and TNF-*α* by ELISA (Figures 3(a) and 3(b)). According to the results, the levels of IL-6 and TNF-*α* were all increased after hemorrhagic shock and resuscitation. However, compared with the Shock+NS group, the levels of the proinflammatory cytokines were lower in the Shock+MRS group. At the tissue level, MRS also significantly decreased the cytokine profile after resuscitation in the cardiac, liver, and kidney tissue samples (Figures 4(a)–4(f)), which confirmed the anti-inflammation effect of MRS. All these results showed that MRS has a very strong inflammatory suppression effect.

### 3.4. Antioxidative Effects of Methane-Rich Saline on Hemorrhagic Shock and Resuscitation

**Figure 5:**
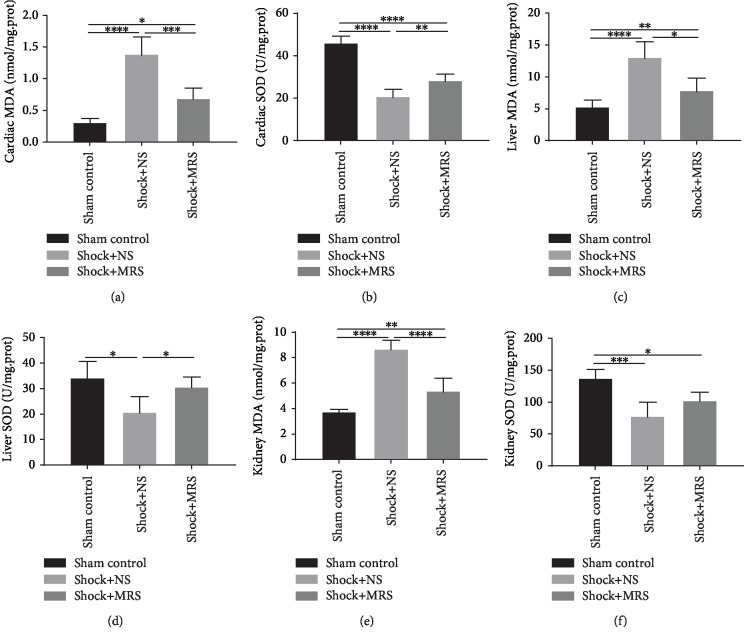
Methane-rich saline attenuated oxidative stress caused by hemorrhagic shock and resuscitation. The levels of (a) MDA and (b) SOD in cardiac tissue, (c) MDA and (d) SOD in liver tissue, and (e) MDA and (f) SOD in kidney tissue were assessed. ^∗^*P* < 0.05, ^∗∗^*P* < 0.01, ^∗∗∗^*P* < 0.001, and ^∗∗∗∗^*P* < 0.0001.

To determine whether MRS exerted an antioxidative effect, the acquired tissue samples were assayed for MDA and SOD activity. Induction of hemorrhagic shock and resuscitation resulted in significantly increased MDA levels in the liver, kidney, and cardiac tissues; however, MDA levels in the Shock+MRS group were lower than those in the Shock+NS group (Figures 5(a), 5(c), and 5(e)). Moreover, although SOD activity was decreased by the induction of hemorrhagic shock and resuscitation, SOD activity in the Shock+MRS group was greater than that in the Shock+NS group (Figures 5(b), 5(d), and 5(f)). These results indicate that MRS exerted an antioxidative effect during hemorrhagic shock and resuscitation.

### 3.5. Antiapoptotic Effects of Methane-Rich Saline on Hemorrhagic Shock and Resuscitation

**Figure 6:**
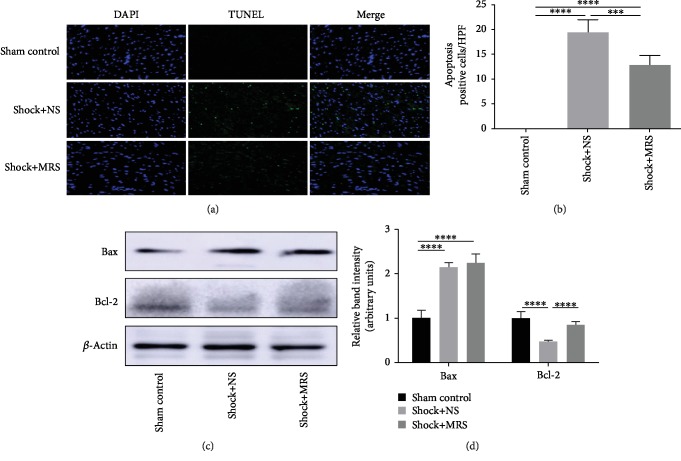
Antiapoptotic effects of methane-rich saline on hemorrhagic shock and resuscitation. (a) TUNEL assay was performed on cardiac tissue slices. (b) The quantity of TUNEL-positive cells was counted in a high-power field. (c) Expression levels of Bcl-2 and Bax in cardiac tissues were evaluated by western blot. (d) The relative band intensities (fold of the sham group) were shown. Representative sections; original magnification, 200x. ^∗^*P* < 0.05, ^∗∗^*P* < 0.01, ^∗∗∗^*P* < 0.001, and ^∗∗∗∗^*P* < 0.0001.

We examined cellular apoptosis in cardiac tissues using TUNEL labeling (Figure 6(a)), with two observers counting the number of TUNEL-positive cells (Figure 6(b)). A significantly increased number of apoptosis-positive cells were observed in the Shock+NS group compared with those found in the sham control group. However, the Shock+MRS group showed a marked reduction in the number of TUNEL-positive cells compared with the Shock+NS group. In agreement with the results of the TUNEL assay, induction of hemorrhagic shock and resuscitation also generated a significant increase in the levels of Bax and a decrease in expression of Bcl-2; treatment with MRS increased the level of Bcl-2 (Figures 6(c) and 6(d)). These results showed that treatment with MRS can dramatically reduce apoptosis after the induction of hemorrhagic shock and resuscitation.

### 3.6. Methane-Rich Saline Induced the PGC-1*α*/SIRT3/SOD2 Signaling Pathway on Hemorrhagic Shock and Resuscitation

**Figure 7:**
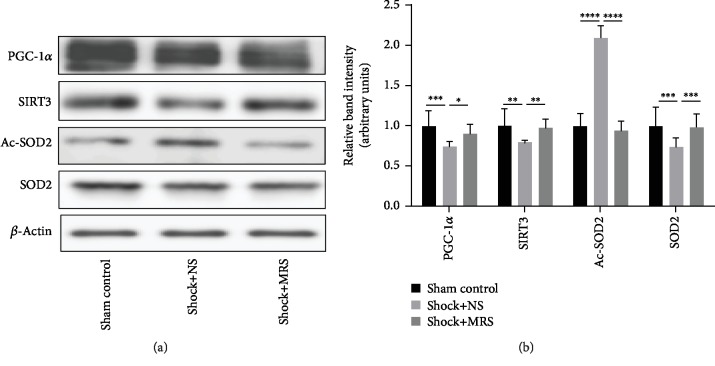
Sirt3 signaling is involved in MRS-mediated protection during hemorrhagic shock and resuscitation. (a) Protein expression of components of the PGC-1*α*/SIRT3/SOD2 signaling pathway (PGC-1*α*/SIRT3/SOD2/Ac-SOD2) was detected by western blotting. (b) Relative band intensities (fold of the sham group) are shown. ^∗^*P* < 0.05, ^∗∗^*P* < 0.01, ^∗∗∗^*P* < 0.001, and ^∗∗∗∗^*P* < 0.0001.

We used western blotting to examine the levels of PGC-1*α*/SIRT3/SOD2 in cardiac tissues after the induction of hemorrhagic shock and resuscitation (Figures 7(a) and 7(b)). Our results show that treatment with MRS restored the expression of SIRT3, which was decreased by hemorrhagic shock and resuscitation; this restoration of SIRT3 levels likely occurred via upregulated expression of PGC-1*α*. In agreement with increased expression of PGC-1*α* and Sirt3, we also observed elevated expression of SOD2 and decreased levels of acetylated SOD2 (Ac-SOD2) (Figures 7(a) and 7(b)), indicating the potential involvement of the PGC-1*α*-Sirt3-SOD2 pathway. These results suggest that treatment with MRS had a positive effect on the expression of the PGC-1*α*/SIRT3/SOD2 signaling pathway.

## 4. Discussion

Hemorrhagic shock and subsequent recovery (occurring at the initial stage of treatment) can lead to ischemia/reperfusion injury (IRI), generating various cell signaling events and exacerbating oxidative stress and inflammatory reactions. This cascade can directly or indirectly promote cell death and ultimately lead to multiple organ dysfunction. Developing therapies that rapidly repair the injury, restore blood volume, improve perfusion pressure, and mitigate the ischemia/reperfusion injury after hemorrhagic shock is critical for preventing organ dysfunction and improving survival.

Methane-rich saline (MRS) was prepared by combining methane and normal saline (NS) under high pressure. MRS has the same osmotic pressure as NS and can replace it as a resuscitation fluid. Previous studies have shown that MRS exerts antioxidative, anti-inflammatory, and antiapoptotic effects in many animal models [5]. In this study, for the first time to our knowledge, we used MRS as a resuscitation fluid to assess its protective ability and explore the mechanisms underlying this protective activity.

Methane and hydrogen gas are products of bacterial metabolism in the human gut; hydrogen can be converted to methane in the gut by certain methane-producing bacteria [11]. Hydrogen-rich solutions can dramatically reduce plasma levels of IL-6, TNF-*α*, MDA, and MPO and upregulate the levels of IL-10 and SOD. This indicates that hydrogenated solutions possess robust anti-inflammatory activity [12]. However, few studies have examined the effects of methane with respect to organ dysfunction induced by hemorrhagic shock and resuscitation. The clinical properties of methane suggest that it can influence several pathological processes including inflammation, oxidation, and apoptosis [13, 14]. However, the mechanisms behind these effects are complex and require further investigation. Our previous study has shown that methane exhibits an antiapoptotic effect by reducing Endoplasmic Reticulum (ER) stress. ER stress can deteriorate cellular functions and trigger CHOP-mediated apoptosis, activating the caspase-12 and Bcl-2/Bax cascade in irreversibly injured cells [15].

We established a hemorrhagic shock and resuscitation rat model to examine the protective effect of MRS. First, we evaluated the MAP of all groups after bleeding and finished resuscitation to confirm the establishment of a hemorrhagic shock and resuscitation model. No differences in MAP were detected between the two shocked groups at any time point, indicating that 1 : 1 resuscitation with blood and MRS performed as well in restoring blood volume and improving perfusion pressure. IRI secondary to hemorrhagic shock and subsequent recovery can cause inflammatory response, oxidative stress, and cell apoptosis, eventually leading to multiple organ damage [16]. Methane was observed to protect against ischemia-reperfusion injury by inhibiting inflammation, suppressing apoptosis, and attenuating oxidative stress in different animal models [6–8, 13]. In this study, we assessed the levels of inflammatory response and oxidative stress among the different groups and found a significantly decreasing trend in the MRS-treated group, suggesting that treatment with MRS exerted strong anti-inflammatory and antioxidative effects. As a recognized antiapoptotic protein, Bcl-2 can prevent the formation of the Bax-induced cascade [17]. In our study, methane significantly enhanced the upregulation of Bcl-2 but did not reduce the level of Bax. So, we speculated that methane exerts antiapoptotic effects mainly by upregulating the expression of Bcl-2.

A key mechanism of IRI is associated with excessive oxidative stress, in which the mitochondrial respiratory chain is the main source of destructive reactive oxygen species (ROS) [18]. Previous reports have found that CH4-mediated intracellular signaling is closely related to mitochondria [19]. Sirt3 is a NAD+-dependent protein deacetylase that is exclusively localized in the mitochondria. Sirt3 regulates the activities of mitochondrial enzymes and processes involved in energy metabolism and is well known as a ROS scavenger and antiapoptotic factor. Activation of Sirt3 mediates the beneficial effects of various agents that protect against myocardial ischemia/reperfusion [20–22]. In addition, Sirt3 protects tissues and cells from hemorrhagic shock by inhibiting cellular oxidative stress, necrosis, and apoptosis and alleviating mitochondrial damage and dysfunction [23–25]. In the present study, the results of western blotting showed that MRS significantly increased Sirt3 expression after HS/R. Mitochondrial manganese superoxide dismutase (SOD2) is essential for detoxification of O2−, which is formed as a byproduct of oxidative phosphorylation in the mitochondria [26]. Reduction and deacetylation of SOD2 by Sirt3 regulates the enzymatic activity of SOD2. In this study, we observed increased SOD2 expression and decreased expression Ac-SOD2 in MRS-treated tissues [27]. PGC-1*α* is a transcription coactivator that plays a key role in regulating mitochondrial biogenesis and energy metabolism in multiple organs [28, 29]. Previous studies in muscle cells, hepatocytes, and neurons found that PGC-1*α* stimulates Sirt3 expression by binding to the Sirt3 promoter region [30]. The data obtained in this study show that the tissues of MRS-treated animals had a higher expression of PGC-1*α* than the controls. These results suggest that the protective effects of MRS are mediated by the PGC-1*α*-Sirt3-SOD2 pathway.

While the results were encouraging, our study still had some limitations. Firstly, we conducted an animal model to perform our experiment; however, in vivo environment was always complex and hard to regulate as purpose; in this condition, more in vitro experiments might need to be done to reveal the mechanism. Secondly, physiological status of anaesthetized animals and normal animals might be different, but it was necessary to anaesthetize animals in order to perform the operation. Moreover, we constructed a control group to minimize the confounding factor brought by anesthesia; we believe it was reasonable not to ignore the effect of anesthesia. Finally, the monitoring time was short, and it was chosen according to common practice. The optimal timing might need to be further investigated, and we could perform other experiments to find the most suitable time in the future.

Recently, the investigation of methane was conducted on ischemia-reperfusion damage and diseases like sepsis, hepatitis, and pancreatitis. Our research could offer evidence to expand the application area of methane medicine in hemorrhagic shock and verify the MRS as a novel potential resuscitation fluid for hemorrhagic shock. Methane is nontoxic and can penetrate cell membranes, and methane is relatively stable. Therefore, methane has great clinical application prospects. However, before we can conduct methane to clinical usage, more research work needs to be performed to establish criteria for optimized dose and timing selection, to conduct delivery methods, and to reveal its shortcomings.

## 5. Conclusions

The results of our present study demonstrate that MRS alleviated organ dysfunction during hemorrhage shock and resuscitation. This likely occurred because MRS inhibited the expression of proinflammatory cytokines, decreased oxidative stress and apoptosis, and facilitated the expression and activity of SOD2 by activating the PGC-1*α*-SIRT3 signaling pathway. These findings suggest that MRS is a promising resuscitation fluid for patients with hemorrhage shock.

## Data Availability

The data related to rat model data, serum cytokine levels, histological staining, and western blot images used to support the findings of this study are available from the corresponding authors upon request.
